# Multidrug-Resistant Uropathogens Causing Community Acquired Urinary Tract Infections among Patients Attending Health Facilities in Mwanza and Dar es Salaam, Tanzania

**DOI:** 10.3390/antibiotics11121718

**Published:** 2022-11-29

**Authors:** Vitus Silago, Nyambura Moremi, Majigo Mtebe, Erick Komba, Salim Masoud, Fauster X. Mgaya, Mariam M. Mirambo, Helmut A. Nyawale, Stephen E. Mshana, Mecky Isaac Matee

**Affiliations:** 1 Department of Microbiology and Immunology, Weill Bugando School of Medicine, Catholic University of Health and Allied Sciences, P.O. Box 1464, Mwanza 33109, Tanzania; 2 Community Development, Gender, Elderly and Children, Ministry of Health, Dar es Salaam 11101, Tanzania; 3 SACIDS Africa Centre of Excellence for Infectious Diseases, Sokoine University of Agriculture, P.O. Box 3297, Morogoro 67125, Tanzania; 4 Department of Microbiology and Immunology, School of Medicine, Muhimbili University of Health and Allied Sciences, P.O. Box 65001, Dar es Salaam 11103, Tanzania; 5 Department of Veterinary Microbiology, Parasitology and Biotechnology, College of Veterinary Medicine and Biomedical Sciences, Sokoine University of Agriculture, Morogoro 67125, Tanzania

**Keywords:** antimicrobial resistance, community acquired urinary tract infections, multidrug resistant bacteria, surveillance, uropathogens

## Abstract

In low-income countries, the empirical treatment of urinary tract infections (UTIs) without laboratory confirmation is very common, especially in primary health facilities. This scenario often leads to unnecessary and ineffective antibiotic prescriptions, prompting the emergence and spread of antimicrobial resistance. We conducted this study to examine the antibiogram of uropathogens causing community-acquired urinary tract infections among outpatients attending selected health facilities in Tanzania. Method: This was a cross-sectional health centre-based survey conducted for a period of five months, from July to November 2021, in the Mwanza and Dar es Salaam regions in Tanzania. We enrolled consecutively a total of 1327 patients aged between 2 and 96 years with a median [IQR] age of 28 [22–39] from Dar es Salaam (n = 649) and Mwanza (n = 678). Results: Significant bacteriuria was observed in 364 (27.4% [95%CI: 25.0–29.9]) patients, from whom 412 urinary pathogens were isolated. Gram-negative bacteria contributed to 57.8% (238) of the 412 uropathogens isolated, of which 221 were Enterobacterales, and *Escherichia coli* was the most frequent. *Staphylococcus aureus* and *Staphylococcus haemolyticus* were the most frequently isolated among Gram-positive uropathogens (n = 156). Generally, resistance among *Escherichia coli* ranged from 0.7% (meropenem) to 86.0% (ampicillin) and from 0.0% (meropenem) to 75.6% (ampicillin) in other Enterobacterales. Moreover, about 45.4% (108) of Enterobacterales and 22.4% (35) of Gram-positive bacteria were multidrug resistant (MDR), *p* = 0.008. We observed 33 MDR patterns among Gram-negative bacteria, predominantly AMP-CIP-TCY (23/108; 21.3%), and 10 MDR patterns among Gram-positive bacteria, most commonly CIP-GEN-TCY (22/35; 62.9%). Conclusion: the presence of a high number of wide-ranging uropathogens that are multidrug resistant to a variety of antibiotics points to the need to strengthen the laboratory diagnostic systems for the regular surveillance of the antimicrobial resistance of uropathogens to guide and update empirical treatment guidelines.

## 1. Introduction

Urinary tract infections (UTIs) are among the most common bacterial infections encountered in healthcare community settings and are associated with increased treatment cost, morbidity, and mortality [1,2]. UTIs can be categorized based on how the infection was acquired, which includes hospital-acquired and community acquired urinary tract infections [1,3,4]. Community acquired urinary tract infections occur when a patient develops a UTI before admission to the healthcare facility and not within 10 days after the patient has been discharged from the healthcare facility [1,5]. *Escherichia coli* is the most common bacterium reported to cause urinary tract infections, while other common uropathogens isolated from urinary tract infections include *Klebsiella* spp., *Staphylococcus* spp., *Enterococcus* spp., *Enterobacter* spp., and *Citrobacter* spp. [1,3]. Community acquired UTIs are usually uncomplicated, as opposed to hospital-acquired UTIs, which, in most cases, are complicated and associated with risk factors such as catheterization and recent antibiotic use [6]. 

In most resource-limited health facilities, community acquired urinary tract infections are the predominant type of UTI and are inappropriately treated with antibiotics due to a lack of laboratory services, as well as sufficiently trained medical personnel [7,8]. Inadvertently, this leads to the emergence and spreading of multidrug-resistant (MDR) bacteria strains in the community [8], leading to recurrences [2,6,7,8] and complications such pyelonephritis with sepsis and pre-term birth in pregnancy [9,10]. In Tanzania, most of the antibiotics’ profile data on UTIs are from patients attending referral hospitals where microbiological services are available [11]. Unfortunately, limited data on UTIs in communities, coupled with a lack of antibiotic stewardship and an absence of laboratory services, leads to irrational uses of antibiotics, especially the widely available cheap medicines, which are often of variable quality [12,13]. 

We designed this cross-sectional health centre-based study to determine the antibiotic susceptibility profiles of multidrug resistant (MDR) bacteria causing community acquired urinary tract infections in Tanzania, where such information is essentially non-existent. Data emanating from this study may be used in drafting evidence-based empirical treatment guidelines among outpatients attending healthcare facilities where urine culture is currently not feasible.

## 2. Results

### 2.1. Patients’ Socio-Demographic and Clinical Characteristics 

This study recruited 1327 patients, aged between 2 and 96 years, with a median age of 28 [IQR: 22–39] years. A majority of patients were females (82.8% (1099/1327)), married (77.9% (965/1327)), and pregnant women (32.8% (435/1327)), and a significant number reported using tap water (68.1% (904/1327)). On the other hand, 44.7% (593/1327) had a previous history of UTI, and 30.8% (403/1327) had used antibiotics within three months before being enrolled in the study; moreover, 35.5% had prescriptions of antibiotics during their enrolment in the study (Table 1).

### 2.2. Prevalence of Causing Community Acquired Urinary Tract Infections and Distribution of Causative Pathogens

Of the 1327 cultured urine samples, 364 had significant microbial growth, which gave an overall prevalence of 27.4% [95%CI: 25.0–29.9] for causing community acquired urinary tract infections. Forty-eight samples had significant dual uropathogens, making a total of 412 uropathogens isolated. Gram-negative bacteria accounted for 57.8% (238/412). Specifically, Mwanza recorded a prevalence of 26.5% [95%CI:.23.2–30.0] while Dar es Salaam recorded a prevalence of 28.4% [95%CI: 24.9–31.9]. Generally, E. coli was the most frequently isolated Gram-negative uropathogen, comprising 66.4% (158/238), whereas S. aureus 20.5% (32/156) and S. haemolyticus 20.5% (32/156) were the most frequently isolated Gram-positive uropathogens, and the other 18 were yeasts (*Candida* spp.) (Table 2).

### 2.3. Percentages of Antibiotic-Resistant Uropathogens Causing Community Acquired Urinary Tract Infections

The overall percentages antibiotic resistance among Gram-negative bacteria ranged from 0.7% (meropenem) to 86.0% (ampicillin) in *E. coli* and from 0.0% (meropenem) to 75.6% (ampicillin) in other Enterobacterales. Antibiotic resistance for non-Enterobacterales ranged from 0.0% (meropenem) to 45.5% (tetracycline) (Table 3). For Gram-positive bacteria, percentages of antibiotic resistance ranged from 2.8% (linezolid) to 84.5% (trimethoprim–sulfamethoxazole) in CoNS, ranged from 4.6% (linezolid) to 86.4% (tetracycline) in *Streptococcus* spp., ranged from 14.3% (ampicillin) to 89.3% (erythromycin) in *Enterococcus* spp., and ranged from 20.0% (linezolid) to 73.4% (erythromycin) in *S. aureus* (Table 4).

### 2.4. Prevalence and Patterns of MDR Bacteria Causing Community Acquired Urinary Tract Infections

The prevalence of MDR bacteria causing community acquired urinary tract infections was significantly high among Gram-negative bacteria than in Gram-positive bacteria (45.4% (108/238) vs. 22.4% (35/156), *p* = 0.008). We observed 33 MDR patterns among Gram-negative bacteria with the predominance of AMP-CIP-TCY (23/108; 21.3%). MDR patterns included resistance towards three (63.6%), four (27.3%), five (6.1%), and six (3.0%) classes of antibiotics (Table 5). We observed 10 MDR patterns among Gram-positive bacteria with the predominance of CIP-GEN-TCY (22/35; 62.9%). The MDR patterns among Gram-positive bacteria included resistance towards three, 85.7% (30/35), and four, 14.3% (5/35), classes of antibiotics (Table 6).

### 2.5. Prevalence and Types of MDR Phenotypes Causing Community Acquired Urinary Tract Infections

**Figure 1:**
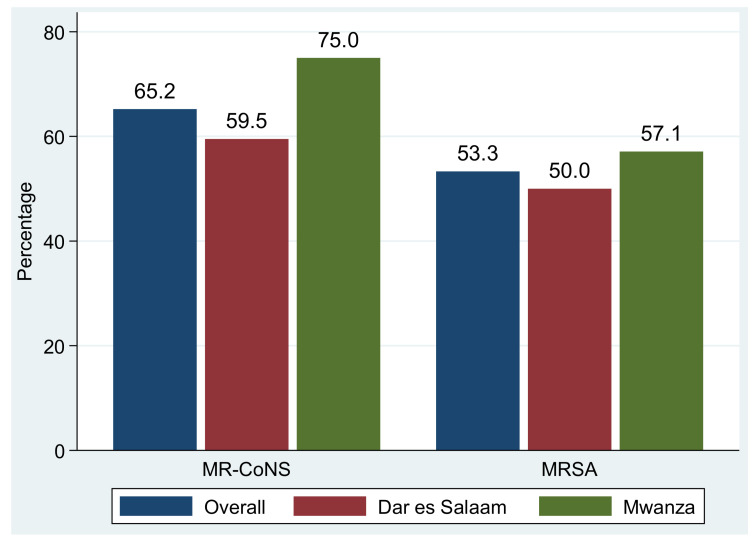
Proportions of methicillin resistance among *S. aureus* (MRSA) and CoNS (MR-CoNS).

**Figure 2:**
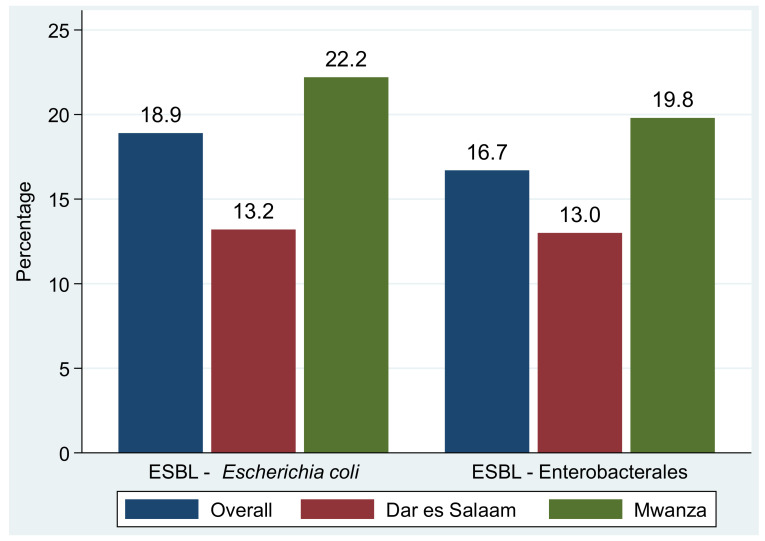
Proportions of extended-spectrum β-lactamase production in *E. coli* and other Enterobacterales.

The proportions of methicillin-resistant *S. aureus* (MRSA) and methicillin-resistant CoNS among *S. aureus* and CoNS causing community acquired urinary tract infections were 53.3% (16/30) and 65.2% (43/66), respectively (Figure 1). The overall prevalence of extended-spectrum β-lactamase (ESBL) production among Enterobacterales was 16.7% (37/221), of which 13% (13/100) was reported in isolates from Dar es Salaam and 19.8% (24/121) was reported from Mwanza. In particular, about 18.9% (27/143) of *E. coli* were ESBL producers, of which Mwanza reported a prevalence of 22.2% (20/90) while Dar es Salaam reported a prevalence of 13.2% (7/53) (*p* = 0.304) (Figure 2).

## 3. Discussion

This study aimed to determine the antibiotic resistance profiles of uropathogens causing community acquired urinary tract infections among outpatients attending selected health centres in Tanzania. This study represents patients from the community with signs and symptoms of UTI from the two largest cities in Tanzania. These data are important because, currently, there is a lack of antimicrobial resistance (AMR) data regarding community infections. The fact that multidrug-resistant, gram-negative, and gram-positive bacteria is the predominant cause of community acquired UTI highlights the coordinated effort required to address the AMR problem in Tanzania and other low-income countries through improved quality healthcare provision.

In the current study, the overall prevalence of community acquired urinary tract infections was 27.4%, with site-specific prevalence rates being 26.5% in Mwanza and 28.4% in Dar es Salaam. Our observed prevalence is slightly lower compared to another study in Tanzania, reporting a prevalence of community acquired urinary tract infections of 38.5% [14]. Similarly, a higher prevalence of community acquired urinary tract infections (39.1%) was reported in Uganda [3]. However, our prevalence matches the prevalence of 26.7% observed in a study in Senegal [15]. These variations could be attributable to differences in antibiotic usage, age, and gender, as well as in the handling and processing of urine samples [16].

Notably, more than one half of isolated uropathogens causing community acquired urinary tract infections were Gram-negative bacteria, of which *E. coli* was frequently isolated, which is in keeping with community acquired urinary tract infection studies conducted in Tanzania [14], Uganda [3], India [17], and Senegal [15]. Besides *E. coli*, *Enterococcus* spp., *S. aureus* and *K. pneumoniae* were the second, third, and fourth most frequent causes of community acquired urinary tract infections in our setting, which is in keeping with findings reported elsewhere [18,19]. Interestingly, other uropathogens included coagulase negative *Staphylococci* (CoNS) such as *S. haemolyticus* (n = 32), *S. epidermidis* (n = 20), and *S. hominis* (n = 3), which have also been reported in previous studies [14,19,20]. 

Similar to previous reports [21,22,23,24,25], we also found uropathogens that are rarely associated with UTI, including *Corynebacterium aurimucosum*, *Corynebacterium striatum*, *Escherichia hermannii*, *Stenotrophomonas maltophilia*, *Mammaliicoccus sciuri* (formerly *Staphylococcus sciuri*), *Acidovorax temperans*, and *Comamonas testosterone* (formerly *Pseudomonas testosterone*); moreover, *A. temperans*, *S. maltophilia*, and *M. sciuri* were isolated in Dar es Salaam only, whereas *C. testosterone* was isolated in Mwanza only. Collectively, these findings indicate that the spectrum of potential uropathogens is wide, warranting the need to employ advanced molecular studies, such as whole-genome sequencing, to screen for their pathogenic and virulence factors. For example, previously, CoNS, *Corynebacterium* spp., and *C. testosterone* were considered skin flora and/or possible laboratory contaminants, while *M. sciuri* was considered to colonize animals; however, increasingly, the bacteria have become associated with opportunistic infections in humans, including UTIs [22,23,26].

Overall, antibiotic resistance of Gram-negative bacteria was less (<30%) to carbapenems, third generation cephalosporins, gentamicin, nitrofurantoin, and trimethoprim–sulfamethoxazole. However, we observed variations in the percentages of the resistance of bacteria pathogens to different antibiotics between the Dar es Salaam and Mwanza samples. For example, *E. coli* isolated from Dar es Salaam showed resistance of 15.1% and 0.0% towards nitrofurantoin and carbapenems, while those isolated from Mwanza showed a resistance of 38.9% and 1.1% towards nitrofurantoin and carbapenems, respectively. Further, other Enterobacterales, excluding *E. coli*, exhibited a resistance of 61.7%, 44.7%, and 27.7% to ampicillin, ciprofloxacin and gentamicin, respectively, in Dar es Salaam, whereas the resistance of 96.8%, 22.6%, and 9.7% towards ampicillin, ciprofloxacin, and gentamicin in respective was observed in Mwanza. 

On the other hand, Gram-positive bacteria, particularly *S. aureus* isolated in the current study, generally showed less resistance to nitrofurantoin (26.6%) and linezolid (20.0%); *Enterococcus* spp. showed less resistance towards ampicillin (14.3%); coagulase negative *Staphylococci* (CoNS) exhibited less resistance to linezolid (2.8%) and nitrofurantoin (16.9%); and *Streptococcus* spp. had low resistance to linezolid (4.6%) and clindamycin (9.1%). Similar to Gram-negative bacteria, differences in the percentage of the resistance of Gram-positive bacteria to different antibiotics were observed between Dar es Salaam and Mwanza. For instance, 43.8%, 31.3%, and 43.8% of *S. aureus* isolated from Dar es Salaam were resistant to ciprofloxacin, clindamycin, and nitrofurantoin compared to 78.6%, 71.5%, and 7.1% of the *S. aureus* isolated from Mwanza towards similar antibiotics, respectively. 

The possible reasons for this observation, namely, the variations in the frequencies of antibiotics’ resistance between the two regions, which are about 1100 km apart, could be due to differences in the drivers responsible for the emergence and spreading of MDR pathogens. These drivers include, but are not limited to, the misuse of antibiotics in clinics, the community, and farms, as well as sanitation infrastructures and waste disposal [27]. Indeed, a previous study demonstrated a clear link between widespread irrational use of antibiotics in the community and the subsequent consecutive induction of resistance [28].

From our observations in the general antibiotic susceptibility profiles of uropathogens isolated during this study, nitrofurantoin, gentamicin, and third generation cephalosporins may be prescribed for Gram-negative bacteria, whereas nitrofurantoin, clindamycin, and linezolid may be prescribed for Gram-positive bacteria. These antibiotics are effective since they are rarely prescribed compared with ampicillin and tetracycline, which are cheaper and widely used [12,13].

The high rate of resistance shown by *E. coli* and other Enterobacterales against ciprofloxacin is concerning since this antibiotic is listed as the first line for an uncomplicated UTI, which is largely caused by Enterobacterales, predominantly *E. coli* [11]. Ciprofloxacin is overly prescribed not only for UTI, but also for many other bacterial infections, often without prescription or proper diagnosis [14,29].

Finally, we would like to acknowledge as a limitation the fact that, for some isolates, there may be a lack of differences in antibiotic resistance between the two regions due to the small sample, such as, for example, in the resistance to ciprofloxacin, clindamycin, and nitrofurantoin for *S. aureus* isolated in Dar es Salaam, which occurred in only 16 cases (7, 5, and 7 cases, respectively), and for n = 14 in Mwanza (11, 10, and 1 cases, respectively). The comparison of these specific isolates does not include many people. 

## 4. Materials and Methods

### 4.1. Study Design and Setting

This cross-sectional health centre-based survey was conducted for a period of five months, from July to November 2021, in the Mwanza and Dar es Salaam regions. Selected health centres were primary health facilities located within 50 km from a reference laboratory. Reference laboratories included clinical microbiology laboratories at the Catholic University of Health and Allied Sciences (CUHAS) in Mwanza and Muhimbili University of Health and Allied Sciences (MUHAS) in Dar es Salaam.

### 4.2. Study Population 

The study consecutively enrolled outpatients at four selected facilities, namely, 2 in Dar es Salaam and 2 in Mwanza. The study involved children aged from 2 years and adults, including pregnant women, who had symptoms of uncomplicated UTI. Patients who resided within the study area and attended one among the selected health centre were eligible for enrolment. All outpatients presenting with signs and symptoms of UTIs [30] with no history of hospital admission in the past 3 months were requested to participate. The symptoms of UTI include frequent urination, pain during urination, cloudy or foul-smelling urine, and pelvic pain.

### 4.3. Data and Sample Collection 

The AfyaData application version 1.4, an open-source tool for collecting and submitting data, was used for collection of data on socio-demographic, behavioural, and clinical aspects. Thereafter, about 5–10 mL of clean-catch, mid-stream urine (MSU) samples were self-collected in a sterile urine container after appropriate instructions were provided to the participants. Samples were transported in a cool box at temperatures of between 4 and 8 ^0^C to CUHAS and MUHAS Reference Clinical Microbiology Laboratories for processing within 2 h of collection.

### 4.4. Laboratory Procedures 

#### 4.4.1. Quantitative Urine Culture 

A 1 μL sterile disposable loop was used for quantitative inoculation of urine samples on plates of 5% sheep blood supplemented Columbia Blood agar (BA; Oxoid, Basingstoke, UK), MacConkey agar with crystal violet (MCA; Oxoid, UK), and Cysteine Lactose Electrolyte Deficient (CLED; Oxoid, UK). Inoculated plates of MCA and CLED were incubated in ambient air, while plates of BA were incubated in a candle jar (5–10% CO_2_) at 35 ± 2 °C for 24 h. Significant microbial growth and colony morphology (e.g., colour, size, and texture) and characteristics on a culture medium (e.g., haemolysis on BA and lactose fermentation on CLED and MCA) were documented. Bacterial counts from ≥10^4^ to ≥10^5^ CFU/mL of no more than two species of microorganisms were considered significant, indicating UTIs, whereas contamination was defined as bacterial counts of ≥10^5^ of more than two species or any growth of <10^4^ CFU/mL [31].

#### 4.4.2. Bacteria Identification 

In-house-prepared conventional biochemical identification tests were used for the preliminary identification of bacteria isolates to their possible species levels as previously described [32,33]. Briefly, bacteria were identified by colonial morphology, Gram stain, and a set of conventional biochemical tests (catalase, oxidase, indole, methyl red, Voges– Proskauer and citrate utilization tests, and lactose fermentation). Moreover, VITEK MS, an automated mass spectrometry microbial identification system that uses Matrix-Assisted Laser Desorption Ionization Time-of-Flight (MALDI-TOF) technology systems, was used to identify some Coagulase-negative *Staphylococci* (CoNS) and miscellaneous Gram-positive rods. 

#### 4.4.3. Antibiotic Susceptibility Testing 

Antimicrobials Susceptibility Testing (AST) was performed using the Kirby–Bauer disk diffusion method [34], and zones of inhibitions were interpreted as recommended by the Clinical and Laboratory Standards Institute (CLSI) guidelines 2021 [35]. Briefly, bacterial colonies from pure culture were transferred to a tube containing 5 mL of sterile 0.9% normal saline and then mixed gently to form a homogenous suspension equivalent to 0.5 McFarland standard solution. A sterile cotton swab was dipped into the bacterial suspension, and the excess fluid was removed by gently pressing and rotating the swab against the inside wall surface of the tube. The swab was then used to inoculate the bacteria evenly over the entire surface of Mueller–Hinton agar (MHA; Oxoid, UK) plate. For *Streptococci* spp., MHA plates supplemented with 5% sheep blood were used and incubated in a candle jar at 35 ± 2 °C for 16–18 h. Other MHA plates were incubated at 35 ± 2 °C in an incubator for 16–18 h. Diameters of the zones of the inhibitions around each antibiotic disk were measured using a Vernier calliper (Mi Tech Metrology, Guangdong, China) in millimetres and were interpreted as sensitive, intermediate, or resistant. Ampicillin (AMP 10 µg; Oxoid, UK), trimethoprim–sulfamethoxazole (SXT 25 µg; Oxoid, UK), tetracycline (TE 30 µg; Oxoid, UK), amoxicillin–clavulanic acid (AMC 30 µg; Oxoid, UK), ciprofloxacin (CIP 5 µg; Oxoid, UK), cefepime (FEP 30 µg; Oxoid, UK), ceftazidime (CAZ 30 µg; Oxoid, UK), ceftriaxone (CRO 30 µg; Oxoid, UK), gentamicin (GEN 10 µg; Oxoid, UK), nitrofurantoin (NIT 300 µg; Oxoid, UK), imipenem (IMP 10 µg; Oxoid, UK), and meropenem (MEM 10 µg; Oxoid, UK) were used to test susceptibility of Gram-negative uropathogens, whereas ampicillin (AMP 10 µg; Oxoid, UK), trimethoprim–sulfamethoxazole (SXT 25 µg; Oxoid, UK), tetracycline (TE 30 µg; Oxoid, UK), ciprofloxacin (CIP 5 µg; Oxoid, UK), gentamicin (GEN 10 µg; Oxoid, UK), nitrofurantoin (NIT 300 µg; Oxoid, UK), clindamycin (CLI 2 µg; Oxoid, UK), erythromycin (ERY 15 µg; Oxoid, UK), cefoxitin (FOX 30 µg; Oxoid, UK; tested for *S. aureus* and CoNS), and linezolid (LNZ 10 µg; Oxoid, UK) were tested for Gram-positive uropathogens. Uropathogens showing resistance to 1 agent in at least 3 different categories of antibiotics were defined as MDR [36].

#### 4.4.4. Quality Control measures

All stains and reagents were clearly labelled, dated, and stored correctly. The operating temperatures of the refrigerator and incubator were monitored and documented daily. All culture media were prepared according to the directions of the manufacturers and were tested for performance and sterility. To standardize the inoculum density of bacterial suspension for the susceptibility test, a 0.5 McFarland standard was used and standard reference strains *S. aureus* (ATCC 25923), MRSA (ATCC 29213), *E. coli* (ATCC 25922), ESBL producing *Klebsiella pneumoniae* (ATCC 700603), and *Pseudomonas aeruginosa* (ATCC 27853) were used as control bacterial strains.

#### 4.4.5. Data Analysis 

EPI INFO statistical software version 7 (CDC, Atlanta, GA, USA) was used for data analysis. Categorical data are presented in percentages and fractions, while continuous data are presented in median [IQR; Interquartile ranges]. The Chi square test was used to determine significance of differences between two proportions. Moreover, WHONET was used to determine the proportions and patterns of MDR among bacteria isolates. A *p*-value of less than 0.05 at a 95% confidence interval (95%CI) was considered statistically significant. 

## 5. Conclusions

Of the 1327 cultured urine samples, 364 had significant microbial growth, which gave an overall prevalence of 27.4% [95%CI: 25.0–29.9] for community acquired UTI. *E. coli* and *Staphylococcus* spp. were the most commonly isolated uropathogens among Gram-negative and Gram-positive bacteria, respectively. About one in two and one in five Gram-negative bacteria and Gram-positive bacteria were MDR strains, respectively. We recommend the continuous AMR surveillance of uropathogens aimed at developing evidence-based empirical treatment guidelines. On the other hand, we recommend further studies to establish the uropathogenic role of CoNS and the other rare isolates such as *Corynebacterium aurimucosum*, *Corynebacterium striatum*, *Escherichia hermannii*, *Stenotrophomonas maltophilia*, *Mammaliicoccus sciuri* (formerly *Staphylococcus sciuri*), *Acidovorax temperans*, and *Comamonas testosterone*. 

## Figures and Tables

**Table 1:** Patients’ socio-demographic and clinical characteristics.

Variables		DAR, *n* = 649	MWANZA, *n* = 678	Overall, *n* = 1327
		*n* (%)	*n* (%)	*n* (%)
Type of patient	Adult males	103 (15.9%)	70 (10.3%)	173 (13.0%)
	Adult females non-pregnant	273 (42.1%)	318 (46.9%)	591 (44.5%)
	Adult females pregnant	195 (30.0%)	240 (35.4%)	435 (32.8%)
	Children	78 (12.0%)	50 (7.4%)	128 (9.7%)
Health centre	Buguruni HC	226 (34.8%)	NA	226 (34.8%)
	Magomeni HC	423 (65.2%)	NA	423 (65.2%)
	Buzuruga HC	NA	338 (49.8%)	338 (49.8%)
	Karume HC	NA	340 (50.2%)	340 (50.2%)
Sex	Male	139 (21.4%)	89 (13.1%)	228 (17.2%)
	Female	510 (78.6%)	589 (86.9%)	1099 (82.8%)
Residency	Urban	649 (100.0%)	338 (49.8%)	987 (74.4%)
	Rural	0 (0.0%)	340 (50.2%)	340 (25.6%)
Median [IQR] age in years		27 [22–38]	28 [23–39]	28 [22–39]
Type of toilet used at home	Pit latrine	144 (22.2%)	106 (15.6%)	250 (18.8%)
	Flush latrine	503 (77.5%)	567 (83.6%)	1070 (80.6%)
	Others	2 (0.3%)	5 (0.7%)	7 (0.5%)
Occupations	Farmer	6 (0.9%)	184 (27.1%)	190 (14.3%)
	Business	259 (39.9%)	174 (25.7%)	433 (32.6%)
	Civil servant	33 (5.1%)	34 (5.0%)	67 (5.0%)
	Housewife	126 (19.4%)	181 (26.7%)	307 (23.1%)
	Not working	137 (21.1%)	49 (7.2%)	186 (14.0%)
	Still on studies	88 (13.6%)	56 (8.3%)	144 (10.8%)
Source of water for domestic use	Tap water	477 (73.5%)	427 (63%)	904 (68.1%)
	Well water	171 (26.3%)	107 (15.8%)	278 (21.0%)
	Lake	1 (0.1%)	144 (21.2%)	145 (10.9%)
Marital status	Single	149 (24.7%)	125 (19.6%)	274 (22.1%)
	Married	454 (75.3%)	511 (80.3%)	965 (77.9%)
Previous history of UTIs	No	263 (40.5%)	461 (68%)	724 (54.6%)
	Yes	379 (58.4%)	214 (31.6%)	593 (44.7%)
	Unknown	7 (1.1%)	3 (0.4%)	10 (0.7%)
Previous antibiotic use past 3 months	Yes	252 (38.8%)	151 (22.2%)	403 (30.4%)
	No	396 (61.2%)	528 (77.8%)	924 (69.6%)
Currently prescribed antibiotic	Yes	322 (49.6%)	149 (22.0%)	471 (35.5%)
	No	327 (50.4%)	529 (78.0%)	856 (64.5%)

DAR = Dar es Salaam, IQR = Interquartile range; NA = not applicable; and UTIs = urinary tract infections.

**Table 2:** Distribution of uropathogens causing community-acquired urinary tract infections.

Variables		Frequencies		
		Mwanza, *n* (%)	Dar es Salaam, *n* (%)	Total, *n* (%)
Culture results	SB	180 (26.5%)	184 (28.4%)	364 (27.4%)
	NMG and NSB	498 (73.5%)	465 (71.6%)	964 (72.6%)
Isolated uropathogens	*E. coli*	90 (43.7%)	68 (33.1%)	158 (38.3%)
	*Enterococcus* spp.	17 (8.3%)	10 (4.9%)	27 (6.6%)
	*S. aureus*	14 (6.8%)	18 (8.7%)	32 (7.8%)
	*K. pneumoniae*	14 (6.8%)	10 (4.9%)	24 (5.8%)
	*S. haemolyticus*	11 (5.3%)	21 (10.2%)	32 (7.8%)
	*S. pyogenes*	11 (5.3%)	2 (0.9%)	13 (3.2%)
	*S. epidermidis*	8 (3.9%)	12 (5.8%)	20 (4.9%)
	*Candida* spp. (yeast)	8 (3.9%)	10 (4.9%)	18 (4.4%)
	*K. aerogenes*	6 (2.9%)	0 (0.0%)	6 (1.5%)
	*C. aurimucosum*	5 (2.4%)	4 (1.9%)	9 (2.2%)
	*A. junii*	0 (0.0%)	5 (2.4%)	5 (1.2%)
	*S. saprophyticus*	0 (0.0%)	5(2.4%)	5 (1.2%)
	Other GNB	14 (6.8%)	23 (11.2%)	37 (8.9%)
	Other GPB	8 (3.9%)	12 (5.8%)	20 (4.9%)
	Total uropathogens	206	206	412

SB = significant bacteriuria; NMG = no microbial growth; NSB = non-significant bacteriuria; GNB = Gram negative bacteria; and GPB = Gram positive bacteria. Other GNB—Mwanza: *Enterobacter kobei* (*n* = 2); *Klebsiella oxytoca* (*n* = 2); *Enterobacter hormaechei* (*n* = 2); *Proteus* spp. (*n* = 2); *Pseudomonas aeruginosa* (*n* = 2); *Citrobacter freundii* (*n* = 1); *Acinetobacter* spp. (*n* = 1); *Comamonas testosterone* (*n* = 1); and *Morganella morganii* (*n* = 1). Dar es Salaam: miscellaneous GNB (*n* = 5), *Klebsiella oxytoca* (*n* = 4), *Acinetobacter* spp. (*n* = 2), *Morganella morganii* (*n* = 2), *Proteus* spp. (*n* = 2), *Acinetobacter schindleri* (*n* = 1), *Acidovorax temperans* (*n* = 1), *Moraxella osioensis* (*n* = 1), *Pseudomonas pasteuri* (*n* = 1), *Citrobacter freundii* (*n* = 1), *Escherichia hermannii* (*n* = 1), *Pseudomonas stutzeri* (*n* = 1), and *Stenotrophomonas maltophilia* (*n* = 1). Other GPB—Mwanza: miscellaneous GPB (*n* = 4); *Streptococcus* spp. (*n* = 2); *Staphylococcus hominis* (*n* = 1); and *Streptococcus agalactiae* (*n* = 1). Dar es Salaam: miscellaneous GPB (*n* = 3), *Streptococcus* spp. (*n* = 3), *Staphylococcus hominis* (*n* = 2), *Streptococcus agalactiae* (*n* = 2), *Mammaliicoccus sciuri* (*n* = 1), and *Corynebacterium striatum* (*n* = 1).

**Table 3:** Percentages resistance of Gram negative uropathogens causing community-acquired urinary tract infections in Tanzania.

Antibiotic	*E. coli*			Other Enterobacterales			Non-Enterobacterales		
	DAR (*n* = 53)	MWZ (*n* = 90)	Overall (*n* = 143)	DAR (*n* = 47)	MWZ (*n* = 31)	Overall (*n* = 78)	DAR (*n* = 14)	MWZ (*n* = 3)	Overall (*n* = 17)
AMP	83.0%	87.8%	86.0%	61.7%	96.8%	75.6%	NA	NA	NA
SXT	81.1%	84.4%	83.2%	61.7%	54.8%	58.9%	25.0% *	50.0% *	30.0% *
TCY	79.3%	74.4%	76.2%	40.4%	54.8%	46.2%	55.6% *	0.0% *	45.5% *
AMC	47.2%	50.2%	48.9%	48.9%	58.1%	52.6%	NA	NA	NA
CIP	50.9%	50.0%	50.4%	44.7%	22.6%	35.9%	14.3%	33.3%	17.7%
FEP	28.3%	28.9%	28.7%	36.2%	22.6%	30.8%	0.0%	33.3%	5.9%
CAZ	28.3%	27.8%	27.9%	31.9%	25.8%	29.5%	35.7%	0.0%	29.4%
CRO	26.4%	27.8%	27.3%	34.0%	25.8%	30.8%	50.0% *	0.0% *	40.0% *
GEN	26.4%	20.0%	22.4%	27.7%	9.7%	20.5%	0.0%	33.3%	5.9%
NIT	15.1%	38.9%	30.1%	40.4%	58.1%	47.4%	NA	NA	NA
IMP	0.0%	1.1%	0.7%	0.0%	0.0%	0.0%	0.0%	0.0%	0.0%
MEM	0.0%	1.1%	0.7%	0.0%	0.0%	0.0%	0.0%	0.0%	0.0%

KEY: DAR = Dar es Salaam, MWZ = Mwanza, AMP = ampicillin, AMC = amoxicillin-clavulanic acid, CAZ = ceftazidime, CRO = ceftriaxone, FEP = cefepime, IMP = imipenem, MEM = meropenem, GEN = gentamicin, CIP = ciprofloxacin, SXT = trimethoprim-sulfamethoxazole, NIT = nitrofurantoin, TCY = tetracycline, NA = not applicable, GNB = Gram negative bacteria, and * *Acinetobacter* spp. only (*n* = 11). Other Enterobacterales included *K. pneumoniae* (*n* = 22), *Klebsiella oxytoca* (*n* = 6), *Enterobacter aerogenes* (*n* = 6), *Enterobacter cloacae* (*n* = 2), unidentified GNR (*n* = 36), *Proteus* spp. (*n* = 4) and *Morganella morganii* (*n* = 2).

**Table 4:** Percentages resistance of Gram positive uropathogens causing community-acquired urinary tract infections in Tanzania.

Antibiotic Agents	*S. aureus*			*Enterococcus* spp.			Coagulase-Negative *Staphylococci* (*CoNS)			*Streptococcus* spp.		
	DAR (*n* = 16)	MWZ (*n* = 14)	Overall (*n* = 30)	DAR (*n* = 10)	MWZ (*n* = 18)	Overall (*n* = 28)	DAR (*n* = 47)	MWZ (*n* = 24)	Overall (*n* = 71)	DAR (*n* = 9)	MWZ (*n* = 13)	Overall (*n* = 22)
AMP	NA	NA	NA	10.0%	16.7%	14.3%	NA	NA	NA	NA	NA	NA
FOX	50.0%	57.1%	53.3%	NA	NA	NA	NA	NA	NA	NA	NA	NA
GEN	37.5%	50.5%	43.3%	NA	NA	NA	44.7%	50.0%	46.5%	NA	NA	NA
CIP	43.8%	78.6%	60.0%	70.0%	50.0%	57.1%	57.5%	62.5%	59.2%	77.8%	69.2%	72.7%
CLI	31.3%	71.5%	50.0%	NA	NA	NA	42.6%	70.8%	52.1%	22.2%	0.0%	9.1%
ERY	68.8%	78.5%	73.4%	90.0%	88.9%	89.3%	80.9%	83.3%	81.7%	55.6%	53.9%	54.6%
NIT	43.8%	7.1%	26.6%	40.0%	27.8%	32.2%	14.9%	20.8%	16.9%	NA	NA	NA
LNZ	18.8%	21.4%	20.0%	40.0%	38.9%	39.3%	4.3%	0.0%	2.8%	0.0%	7.7%	4.6%
TCY	50.1%	64.3%	56.7%	NA	NA	NA	61.7%	66.7%	63.4%	77.8%	92.3%	86.4%
SXT	31.3%	64.3%	46.6%	40.0%	72.3%	60.7%	78.7%	95.8%	84.5%	33.3%	38.5%	36.4%

KEY: DAR = Dar es Salaam, MWZ = Mwanza, AMP = ampicillin, FOX = cefoxitin, GEN = gentamicin, CIP = ciprofloxacin, LI = clindamycin, ERY = erythromycin, NIT = nitrofurantoin, LNZ = linezolid, TCY = tetracycline, SXT = trimethoprim-sulfamethoxazole, NA = not applicable, and *CoNS including all other *Staphylococcus* spp. except *S. aureus* and miscellaneous GPB.

**Table 5:** Patterns of MDR bacteria among Gram negative uropathogens causing community-acquired urinary tract infections in Tanzania.

Isolate	MDR Patterns	Classes Resisted	Frequency		
			Mwanza (*n* = 61)	DAR (*n* = 47)	Overall (*n* = 108)
*E. coli*	CIP-NIT-TCY	3	2	1	3
	CIP-GEN-TCY	3	0	1	1
	AMP-GEN-TCY	3	3	2	5
	AMP-NIT-TCY	3	5	1	6
	AMP-GEN-NIT	3	0	1	1
	AMP-CIP-TCY	3	12	11	23
	AMP-NIT-TCY	3	0	1	1
	AMP-CIP-GEN	3	1	0	1
	AMP-CIP-NIT-TCY	4	13	1	14
	AMP-CIP-GEN-TCY	4	8	8	16
	AMP-CIP-GEN-NIT	4	0	1	1
	AMP-CIP-GEN-NIT-TCY	5	5	1	6
	AMP-CIP-GEN-MEM-NIT-TCY	6	1	0	1
*K. pneumoniae*	AMP-CIP-NIT	3	0	1	1
	AMP-CIP-TCY	3	2	0	2
	AMP-NIT-TCY	3	1	0	1
	AMP-CIP-NIT-TCY	4	3	3	6
	AMP-GEN-NIT-TCY	4	1	0	1
Other GNB	AMP-NIT-TCY	3	1	0	1
	CIP-GEN-TCY	3	0	1	1
	AMP-CIP-GEN	3	0	4	4
	CIP-NIT-TCY	3	0	1	1
	AMP-CIP-GEN-TCY	4	0	2	2
	AMP-CIP-GEN-NIT-TCY	5	0	2	2
*K. oxytoca*	AMP-CIP-TCY	3	1	0	1
	AMP-CIP-NIT	3	0	1	1
	AMP-CIP-GEN	3	0	1	1
	AMP-CIP-GEN-TCY	4	0	1	1
*M. morganii*	AMP-NIT-TCY	3	1	0	1
	GEN-NIT-TCY	3	0	1	1
*E. hormaechei*	AMP-NIT-TCY	3	1	0	1
Total		33	61	47	108

**Table 6:** Patterns of MDR bacteria among Gram-positive uropathogens causing community-acquired urinary tract infections in Tanzania.

Isolate	MDR Patterns	Classes Resisted	Frequency		
			Mwanza (*n* = 18)	DAR (*n* = 17)	Overall (*n* = 35)
*S. aureus*	CIP-NIT-TCY	3	1	1	2
	CIP-GEN-TCY	3	6	2	8
	CIP-GEN-NIT	3	0	1	1
	CIP-GEN-NIT-TCY	4	0	1	1
Other GPC	CIP-NIT-TCY	3	1	0	1
	CIP-GEN-TCY	3	1	0	1
CoNS	CIP-NIT-TCY	3	3	1	4
	CIP-GEN-TCY	3	5	8	13
	CIP-GEN-NIT	3	0	1	1
	CIP-GEN-NIT-TCY	4	1	2	3
Total		10	18	17	35

## Data Availability

The datasets used and/or analysed during the current study are available from the corresponding author on reasonable request.
